# Bilateral renal cortical necrosis in acute pancreatitis

**DOI:** 10.4103/0971-4065.57112

**Published:** 2009-07

**Authors:** G. S. R. Krishna, K. C. Kishore, N. P. Sriram, V. V. Sainaresh, A. Y. Lakshmi, V. Siva Kumar

**Affiliations:** Department of Radiology, Sri Venkateswara Institute of Medical Sciences (SVIMS), Tirupati, Andhra Pradesh-517 501, India; 1 Department of Nephrology, Sri Venkateswara Institute of Medical Sciences (SVIMS), Tirupati, Andhra Pradesh-517 501, India

A 22-year-old male with no premorbid illness presented to emergency with vomiting, peri umbilical abdominal pain with pain radiating to the back following an alcoholc binge. He developed oliguria followed by anuria over two days. On examination, he was hemodynamically stable (BP – 120/80 mm of Hg) and had tenderness in the epigastrium and right hypochondriac areas. Investigations revealed neutrophilic leucocytosis (14200 per *μ*l), severe renal failure (Serum creatinine: 13.4 mg/dl) and elevated pancreatic enzymes (serum amylase: 397 U/L, lipase 210 U/L, normal values being 20-96 U/L and 3-43 U/L respectively), elevated LDH (1802 U/L, normal being 115-221 U/L). Contrast enhanced Computed tomography of the abdomen [Figure 1] revealed diffuse and bilateral cortical hypodense areas surrounded by capsular enhancement in both kidneys, which is characteristic of renal cortical necrosis. He received general supportive management, antibiotics and dialysis support. Patient left the hospital against advice on the third hospital day.

**Figure 1:**
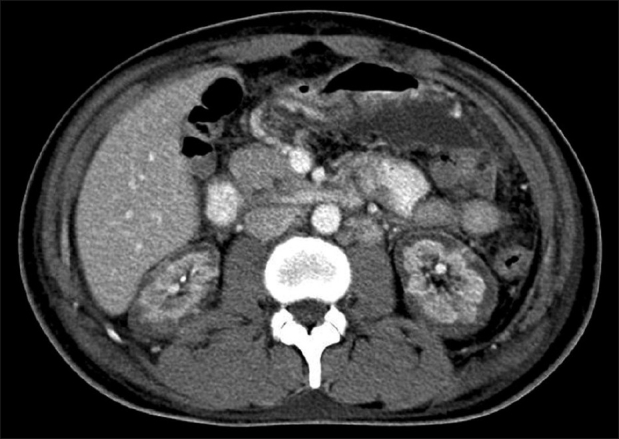
Contrast enhanced CT of the abdomen showing diffuse hypodense areas in the cortex surrounded by capsular enhancement in both the kidneys

## Discussion

Bilateral cortical necrosis is a rare, often irreversible form of acute tubular necrosis. In a study on acute renal failure from north India, the incidence reported was 3.8%.[1] Only eight cases of bilateral cortical necrosis following acute pancreatitis were reported so far in the literature.[2] Cortical necrosis generally results from decreased blood supply within the microcirculation of renal cortex that follows septic shock or volume depletion. However, its cause remains elusive in the presence of normotension. It was ascribed to the release of vasoactive or cytotoxic substances during pancreatitis.[2,3] Interestingly, our patient was normotensive through out. In view of its rarity, this entity is reported.
